# Composition and Antimicrobial Activities of *Lippia multiflora* Moldenke, *Mentha* x *piperita* L. and *Ocimum basilicum* L. Essential Oils and Their Major Monoterpene Alcohols Alone and in Combination

**DOI:** 10.3390/molecules15117825

**Published:** 2010-11-03

**Authors:** Imaël Henri Nestor Bassolé, Aline Lamien-Meda, Balé Bayala, Souleymane Tirogo, Chlodwig Franz, Johannes Novak, Roger Charles Nebié, Mamoudou Hama Dicko

**Affiliations:** 1 Laboratoire BAEBIB, UFR-SVT, Université de Ouagadougou, 09 BP 848 Ouagadougou, Burkina Faso; 2 Institute for Applied Botany and Pharmacognosy, Department of Farm Animal and Public Health in Veterinary Medicine, University of Veterinary Medicine, Veterinärplatz 1, A-1210, Vienna, Austria; E-Mail: aline.lamien-meda@vetmeduni.ac.at (A.L.-M.); 3 Institut de Recherche en Sciences Appliquées et Techniques, Departement de Substances Naturelles 03, BP 7027, Ouagadougou 03, Burkina Faso; E-Mail: neroch@hotmail.com (R.C.N.)

**Keywords:** essential oils, chemical composition, antibacterial activity, synergism

## Abstract

Essential oils from leaves of *Lippia multiflora*, *Mentha x piperita* and *Ocimum basilicum* from Burkina Faso were analysed by GC–FID and GC–MS. Major components were *p*-cymene, thymol, β-caryophyllene, carvacrol and carvone for *L. multiflora*, menthol and *iso*-menthone for *M. x piperita* and, linalool and eugenol for *O. basilicum*. The essential oils and their major monoterpene alcohols were tested against nine bacterial strains using the disc diffusion and broth microdilution methods. The essential oils with high phenolic contents were the most effective antimicrobials. The checkerboard method was used to quantify the efficacy of paired combinations of essential oils and their major components. The best synergetic effects among essential oils and major components were obtained with combinations involving *O. basilicum* essential oil and eugenol, respectively. As phenolic components are characterized by a strong spicy aroma, this study suggests that the selection of certain combinations of EOs could help to reduce the amount of essential oils and consequently reduce any adverse sensory impact in food.

## Introduction

Many food products are perishable by nature and require protection from spoilage during their preparation, storage and distribution to give them desired shelf-life. Therefore, there is a great interest in finding new methods of food preservation with natural compounds. For this purpose essential oils (EOs) are good candidates as antibacterial additives [1]. Several *in vitro* studies have shown a high efficiency of EOs against food-borne pathogens and spoilage bacteria [2].

Most of the antimicrobial activity in EOs appears to derive from oxygenated terpenoids such as alcoholic and phenolic terpenes, while other constituents are believed to contribute little to the antimicrobial effect [3,4]. Although EOs are widely applied as natural antimicrobials, their organoleptic properties may alter the taste of food or exceed acceptable flavour thresholds [5,6]. One solution is the application of EOs or their components in combination. Previous studies have reported that antimicrobial activities of EOs might result to synergistic or antagonistic effects between their major components [7]. Synergistic or antagonistic effects are observed when the activity of the combined product is greater or lower than the sum of individual chemical components, respectively. 

The EOs from *Lippia multiflora* Moldenke, *Mentha x piperita* L. and *Ocimum basilicum* L. have been widely used in food, beverage, cosmetic, health and tobacco industries. Their major components include monoterpene alcohols and phenols, among which are menthol, linalool, thymol, carvacrol, and eugenol [8,9,10]. Inouye *et al.* [11] reported that the major respiratory tract pathogens, including *Haemophilus influenzae*, *Streptococcus pneumoniae, Streptococcus pyogenes* and *Staphylococcus aureus*, were susceptible to *M. x piperita* oil and its major components menthol and menthone. Menthol was the most effective of the peppermint components. Linalool exhibited strong inhibitory effect against 17 bacteria and 10 fungi [12]. 

Carvacrol, eugenol and thymol were able to inhibit the growth of bacteria such as *Staphylococcus aureus* and *Escherichia coli* [3,13]. Paired combinations of eugenol, carvacrol, thymol and menthol have been found to exert synergistic, additive or antagonistic effects, depending on the corresponding microorganism. Pei *et al.* [14] reported synergetic effects of paired combinations of carvacrol, eugenol, thymol against *E. Coli*, whereas Gallucci *et al.* [15] have found synergetic effects of menthol/thymol and thymol/eugenol against *S. aureus* and *B. Cereus*, respectively, when studying paired combination of carvacrol, eugenol, menthol and thymol. To the best of our knowledge, there is no published report regarding the antimicrobial effectiveness of combinations of EOs of *L. multiflora, M. x piperita* and *O. Basilicum,* hence the aim of the present study was to assess the susceptibility of food-borne bacteria to single and paired combinations of the EOs of *L. multiflora, M. x piperita* and *O. basilicum* and their major monoterpene alcohols, to detect synergistic, additive or antagonistic effects and to correlate the chemical composition to the antimicrobial activity. 

## Results and Discussion

### Chemical composition of the essential oil

The total yields of volatile chemicals from *L. multiflora, M. x piperita* and *O. basilicum* were 2.2% (w/w), 1.9% (w/w) and 0.9% (w/w), respectively. Monoterpenes were the most abundant volatiles detected in the EOs (Table 1). Oxygenated terpenes were the most dominant in the EO of *M. x piperita* and *O. Basilicum*, whereas terpene hydrocarbons were the most abundant in the EO of *L. multiflora*. 

*L. multiflora* EO was characterised by the presence of twenty-nine components, representing 97.3% of the total oil. *p*-Cymene (21.3%), thymol (14%), β-caryophyllene (12.9%), carvacrol (9.3%) and carvone (8.6%) were the major constituents. Minor components were 1,8-cineole (5%), α-humulene (3.5%), α-amorphene (3.3%), α-phellandrene (3.1%), β-myrcene (2.4%), α-thujene (2.3%), α-terpinene (1.9%), thymol acetate (1.6%), bicyclogermacrene (1.3%) and δ-cadinene (1.2%). 

**Table 1:** Chemical Composition of *Lippia multiflora, Mentha x piperita* and *Ocimum basilicum* essential oils.

Compounds	RRI^a^	RI^b^	PA^c^		
			*L. multiflora*	*M. x piperita*	*O. basilicum*
α-Thujene	929	930	2.3	-	-
α-Pinene	936	939	0.7	0.7	-
Camphene	952	954	0.2	-	-
β-Pinene	976	979	0.7	0.9	0.2
β-Myrcene	992	991	2.4	-	0.8
α-Phellandrene	1005	1003	3.1	-	-
α-Terpinene	1018	1017	1.9	-	-
*p*-cymene	1025	1025	21.3	-	-
Limonene	1027	1029	-	0.2	-
1,8-Cineole	1032	1031	5.0	4.1	1.7
β-Ocimene	1051	1050	0.9	-	2.7
Carvone	1060	1060	8.6	-	-
γ-Terpinene	1064	1060	-	0.1	-
Fenchone	1087	1087	-	-	0.2
Linalool	1098	1097	-	-	57.0
Terpinolene	1144	1132	0.4	-	-
Camphor	1144	1146	-	-	0.2
Menthone	1154	1153	-	25.2	-
Menthofuran	1163	1164	-	6.8	-
*iso*-menthone	1165	1163	-	5.3	-
Menthol	1174	1172	-	39.3	-
4-Terpineol	1182	1177	0.3	-	-
*iso*-Menthol	1183	1183	-	0.9	-
*neo*-Menthol	1191	1187	-	0.2	-
α-Terpineol	1197	1189	-	-	0.4
α-Fenchyl acetate	1219	1220	-	-	0.4
Pulegone	1242	1237	-	1.4	-
Piperitone	1262	1253	-	0.1	-
Geraniol	1266	1253	-	-	0.8
*neo,iso*-menthyl acetate	1275	1274	-	0.4	-
Thymol	1271	1290	14.0	-	-
Menthyl acetate	1291	1295	-	6.7	-
Thymol acetate	1305	1299	1.6	-	-
*iso*-Menthyl acetate	1307	1305	-	0.3	-
Carvacrol	1352	1352	9.3	-	-
Eugenol	1357	1359	-	-	19.2
Carvacrol acetate	1373	1373	0.4	-	-
α-Copaene	1379	1377	0.5	-	-
β-Elemene	1393	1391	-	-	0.6
Caryophyllene	1422	1409	-	0.8	-
β-Caryophyllene	1422	1419	12.9	-	-
*Trans*-α-Bergamotene	1438	1435	-	-	2.7
α-Humulene	1458	1455	3.5	-	0.2
*allo*-Aromadendrene	1465	1460	0.3	-	-
α-Amorphene	1485	1485	3.3	-	-
β-Cubebene	1485	1485	-	-	0.5
Bicyclogermacrene	1499	1500	-	-	1.0
Germacrene D	1500	1485	0.1	-	-
Germacrene A	1509	1508	-	-	1.1
γ-Cadinene	1517	1513	-	-	1.6
β-Selinene	1527	1490	0.3	-	-
Bicyclogermacrene	1553	1500	1.3	-	-
δ-Cadinene	1587	1523	1.2	-	-
Elemol	1617	1550	0.2	-	-
Caryophyllene oxide	1643	1583	0.2	-	-
α-Cadinol	1649	1652	-	-	3.2
β-Eudesmol	1662	1651	0.3	-	-
Total			97.2	93.4	94.5
Monoterpenes			73.1	92.6	83.6
Sesquiterpenes			24.1	0.8	10.9
Terpenes hydrocarbons			56.4	2.7	8.7
Oxygenated terpenes			40.8	90.7	85.8

a RRI, relative retention indices relative to *n*-alkanes on a DB5 column; b RI, literature retention indices; c PA, peak area expressed in area percentage (%, area).

Seventeen components characterised the EO of *M. x piperita*, representing 93.4% of the total oil. Quantitatively, the most abundant were menthol (39.3%) and menthone (25.2%). Minor components were menthofuran (6.8%), menthyl acetate (6.7%), *iso*-menthone (5.3%), 1,8-cineole (4.1%) and pulegone (1.4%). 

A total of nineteen constituents were identified in the EO of *O. Basilicum*, representing 94.6 % of the total oil. The most abundant compounds were linalool (57%) and eugenol (19.2%). The minor compounds were α-cadinol (3.2%), β-ocimene (2.7%), *trans*-α-bergamotene (2.7%), 1,8-cineole (1.7%), γ-cadinene (1.6%), germacrene A (1.1%) and bicyclogermacrene (1.0%). As seen, the chemical composition of the different essential oils varied greatly from one species to another.

The essential oil of *L. multiflora* was characterized by high levels of *p*-cymene, thymol, β-caryophyllene and carvacrol. This chemical composition is different to those with less than 10% of sesquiterpenes previously described [16,17]. Considering the major constituent, the literature reveals that *L. multiflor*a exhibited intraspecific variation in its oil composition [18,19,20]. However, the high concentrations of *p*-cymene and thymol in this sample make it similar to those found by Bassolé *et al.* [8] and Abena *et al.* [21].

According to Lawrence and Shu [22], a typical American peppermint oil contains mainly α-pinene (1.4%), β-pinene (1.8%), limonene (2.5%), 1,8-cineole (7.3%), *trans*-sabinene hydrate (1.0%), menthone (18.7%), menthofuran (3.0%), *iso*-menthone (2.5%), menthyl acetate (3.6%), *neo*-menthol (3.1%), menthol (40%) and germacrene D (1.3%). In this respect the African peppermint menthol content matched the above data. However, menthone, menthofuran, *iso*-menthone and menthyl acetate contents of the African oil were higher than that of the American peppermint.

On the basis of the oil composition, seven chemotypes of *O. basilicum* essential oil have been described [10]: (1) high-linalool, (2) linalool/eugenol, (3) methyl chavicol without linalool, (4) methyl chavicol/linalool, (5) methyl eugenol/linalool, (6) methyl cinnamate/linalool and (7) bergamotene chemotypes. Our sample belongs to linalool/eugenol chemotype, as in other studies [23].

### Antibacterial activity

The *in vitro* antimicrobial activities of *L. multiflora, M. x piperita* and *O. basilicum* EOs and their major components against the studied microorganisms were qualitatively and quantitatively assessed by the presence or absence of inhibition zones, zone diameters (ZDs) and MIC values. The correlation between two different screening methods examined was generally larger ZDs correlated with lower MICs. 

According to the results, EOs exhibited moderate to strong and, in a few cases, a very weak antimicrobial activity against the tested species (Table 2). However, the EOs of *L. multiflora, M. x piperita* and *O. basilicum* failed to show antibacterial activity against *P. aeruginosa CRBIP 19.249*. 

Results obtained from disc-diffusion method, followed by measurements of MIC values, indicated that the EO of *L. multiflora* was the most effective against *S. aureus* (MIC = 1.2 ± 0 mg/mL), *S. enterica* (MIC = 4.2 ± 0.1 mg/mL) and *S. dysenteria* (MIC = 4.4 ± 0.1 mg/mL). The EO of *O. basilicum* had the lowest MIC for *E. faecalis* (MIC = 4.2 ± 0.1 mg/mL), *E. areogenes* (MIC = 4.2 ± 0.1), *S. typhimurium* (MIC = 5 ± 0 mg/mL) and *E. coli* (MIC = 8.3 ± 0.1 mg/mL). Among EOs, the EO of *M. x piperita* showed the weakest antimicrobial activity. *S. aureus*, *E. faecalis*, *S. enterica* and *S. dysenteria* were the most susceptible to the three EOs whereas *L. monocytogenes* was the least sensitive. 

The antibacterial activity of Eos’ major components have been evaluated against the strains (*L. monocytogenes*, *E. aerogenes*, *E. coli*, *P. aeruginosa*) least susceptible to EOs. Among the five major components of the investigated EOs, thymol and carvacrol demonstrated the strongest antibacterial activity against the tested microorganisms, followed by eugenol, whereas linalool and menthol demonstrated moderate and limited activities, respectively. Major components alone more significantly inhibited test bacteria than EOs.

**Table 2:** Antimicrobial activities of *L. multiflora, M. x piperita* and *O. basilicum* essential oils and their major components.

	*L. multiflora*	*M. x piperita*	*O. basilicum*	Tc	Er	Carvacrol	Eugenol	Linalool	Menthol	Thymol
Diameter of inhibition zone (mm)^A^										
*S. aureus* ATCC 9144	39.3 ± 1.8^a^	27 ± 1.3^ b^	23.7 ± 0.9^ c^	34 ± 0^ d^	30.5 ± 0.5^ e^	nd	nd	nd	nd	nd
*E. faecalis* CIP 103907	22.3 ± 1.8^ a^	24 ± 0^ a^	28.3 ± 1.1^ b^	16 ± 1^ c^	8 ± 0^ d^	nd	nd	nd	nd	nd
*L. monocytogenes* CRBIP 13.134	14.3 ± 1.6^ a^	22.3 ± 0.8 ^b^	19.8 ± 2.3 ^a^	19 ± 0 ^a^	8 ± 0^ c^	34.7 ± 1.8^ d^	26.3 ± 1.1^ a^	10.7 ± 0.4^ a^	9.7 ± 1.8^ a^	56 ± 2.7^ a^
*E. aerogenes* CIP 104725	12 ± 1.5^ a^	12.5 ± 1.5^ a^	28.5 ± 0.8^ b^	21 ± 0 ^c^	0 ± 0	47.7 ± 1.8^ d^	26.3 ± 0.9^ b^	11 ± 0.7^ e^	16.3 ± 2.2^ a^	56 ± 4^ d^
*E. coli* CIP 105182	14 ± 2.5^a^	10.8 ± 1.1^ a^	29.3 ± 0.9^ b^	24 ± 1^c^	10 ± 0^ a^	53 ± 2^ c^	24.3 ± 0.9^ d^	15 ± 0^ a^	8 ± 1.3 ^e^	47 ± 1^ c^
*P. aeruginosa* CRBIP 19.249	0	0	0	0	0	22 ± 1.3^ a^	27.3 ± 0.4^ b^	8.3 ± 0.4^ c^	11 ± 0^ d^	26 ± 1.3^ a^
*S. enterica* CIP 105150	29.5 ± 0.8^ a^	21.3 ± 0.4^ b^	25.3 ± 0.4^ c^	23 ± 1^ b^	31 ± 1^ a^	nd	nd	nd	nd	nd
*S. typhimurium* ATCC 13311	15.5 ± 2.3^ a^	18 ± 0.5^ a^	24.7 ± 0.4^ b^	25 ± 0^ b^	24.5 ± 4.5^ b^	nd	nd	nd	nd	nd
*S. dysenteria* (CIP 54.51)	29 ± 1.3^ a^	26.6 ± 7.7^ b^	22 ± 1.3^ c^	12 ± 0^ d^	9.5 ± 0.5^ e^	nd	nd	nd	nd	nd
Minimum inhibitory concentration (mg/mL)										
*S. aureus* ATCC 9144	1.2 ± 0^ a^	8.3 ± 0.2^ b^	2.5 ± 0^ c^	nd	nd	nd	nd	nd	nd	nd
*E. faecalis* CIP 103907	6.7 ± 0.2^ a^	8.3 ± 0.1^ b^	4.2 ± 0.1^ c^	nd	nd	nd	nd	nd	nd	nd
*L. monocytogenes* CRBIP 13.134	20 ± 0^ a^	10 ± 0^ b^	16.7 ± 0.2^ a^	nd	nd	0.2 ± 0^ c^	1.6 ± 0.2^ d^	6.7 ± 0.2^ e^	16.7 ± 0.4^ a^	0.2 ± 0^ f^
*E. aerogenes* CIP 104725	16.7 ± 0.4^ a^	>80	4.2 ± 0.1^ b^	nd	nd	0.2 ± 0^ c^	1.6 ± 0.2^ d^	4.2 ± 0.1^ b^	11.7 ± 0.6^ e^	0.2 ± 0^ c^
*E. coli* CIP 105182	26.7 ± 0.9^ a^	40 ± 0.3^ b^	8.3 ± 0.1^ c^	nd	nd	0.2 ± 0^ d^	1 ± 0.1^ e^	3.3 ± 0.1^ f^	11.7 ± 0.6^ g^	0.7 ± 0.1^ h^
*P. aeruginosa* CRBIP 19.249	>80	>80	>80	nd	nd	0.3 ± 0^ a^	2.1 ± 0.2^ b^	6.7 ± 0.2^ c^	16.7 ± 0.4^ d^	0.8 ± 0.1^ e^
*S. enteric* CIP 105150	4.2 ± 0.1^ a^	8.3 ± 0.2^ b^	5 ± 0^ c^	nd	nd	nd	nd	nd	nd	nd
*S. typhimurium* ATCC 13311	20 ± 0^ a^	13.3 ± 0.2^ b^	5 ± 0^ c^	nd	nd	nd	nd	nd	nd	nd
*S. dysenteria* CIP 54.51	4.4 ± 0.1^ a^	5.8 ± 0.1^ a^	8.3 ± 0.1^ b^	nd	nd	nd	nd	nd	nd	nd

Data in the same line followed by different letters are statistically different by Fisher’s test (p < 0.05). Values are means ± standard deviation of three separate experiments. ^A^ diameter of inhibition zone (mm) including disc diameter of 6 mm. Er = erythromycin (15 µg/disc); Tc = Tetracycline (30 UI); nd: not determined.

The plant EOs tested in this study exhibited variable antibacterial activities against the eight foodborne bacteria, except *P aeruginosa*. To some extent, these results were similar to those of previous studies [8,24]. The resistance of *P. aeruginosa* to *L. multiflora, M. x piperita* and *O. basilicum* EOs has been already reported [24,25]. 

The antimicrobial activities of the EOs appear to be related to their chemical composition. The greater antibacterial potential of *L. multiflora* oil could be explained by the presence of thymol and carvacrol, which showed very strong antibacterial activity (Table 2). The EO of *O. basilicum* possesses linalool and eugenol as main components, among which eugenol exhibited high antibacterial properties, but lower than carvacrol and thymol. The lowest antibacterial activity of *M. x piperita* could be due to its main component menthol, which showed a weaker activity than eugenol. Previous work has reported a potential antibacterial effect and a similar ranking for carvacrol, thymol, eugenol and menthol, but the author of that work did not find any antibacterial activity with menthol [15]. However, both *M. x piperita* oil and menthol have been shown to be active against a variety of microorganisms [11]. In oils dominated by linalool, a low antibacterial activity has been also reported [3], though, minor components such as carvone, 1,8-cineole, menthone and terpineol can also contribute to the antimicrobial activity of the oil [3,11,26]. 

The lower antibacterial activity of the EOs when compared to their major components could be due to interaction between EO components. Carson and Riley [26] reported inhibitory activity for terpinen-4-ol, but not for the oil of *Melaleuca alternifolia* against *P. Aeruginosa*, and our findings support their suggestion that antagonism could occur between components of the oil. 

The components with phenolic structures, such as carvacrol, eugenol and thymol were highly active against the test microorganisms. The importance of phenolic ring and the hydroxyl group in the phenolic structure in terms of activity have been showed by Dorman and Deans [27] by comparing antimicrobial activity of carvacrol to its methyl ether and to *p*-cymene respectively. 

Several mechanisms of antimicrobial action of terpenes have been described. Wendakoon and Sakaguchi [28] hypothesized that the hydroxyl group on eugenol might react with proteins and preventing enzyme action. Carvacrol and thymol were hydrophobic and prone to disturb the outer membrane of Gram-negative bacteria, releasing lipopolysaccharides, and increasing the permeability of the cytoplasmic membrane to ATP [29,30]. Little is known on the mechanism of action of linalool. 

Since higher concentrations of plant EOs are generally required when added to food, the application of EOs in food may be limited due to the resulting changes in organoleptic and textural quality of food or interactions of EOs with food components [31]. Accordingly, a challenge for practical application of EOs is to develop optimised low dose combinations to maintain product safety and shelf-life, thereby minimising the undesirable flavour and sensory changes associated with the addition of high concentrations of EOs [6]. 

### Interaction studies

The FIC indices ranged from 0.11 to 2.47 for paired combinations of *L. multiflora*, *M. x piperita* and *O. basilicum* EOs (Table 3). All paired combinations had synergetic effects on the inhibition of *E. faecalis*, *L. monocytogenes* and *E. coli*. Combinations of *L. multiflora* with *M. x piperita* or *O. basilicum* had synergetic effects on the inhibition of *S. typhimurium* and *S. dysenteria*. 

**Table 3:** Fractional Inhibitory Concentration (FIC) and interaction between essential oils.

Strains	LM^A^#MP^B^		LM#OB^C^		MP#OB	
	FIC	Interaction	FIC	Interaction	FIC	Interaction
*S. aureus* ATCC 9144	0.85	Ad	2.20	I	0.36	S
*E. faecalis* CIP 103907	0.11	S	0.44	S	0.37	S
*L. monocytogenes* CRBIP 13.134	0.16	S	0.05	S	0.27	S
*E. aerogenes* CIP 104725	ND	ND	0.11	S	ND	ND
*E. coli* CIP 105182	0.19	S	0.22	S	0.29	S
*S. enterica* CIP 105150	2.42	I	0.20	S	2.47	I
*S. typhimurium* ATCC 13311	0.15	S	0.12	S	0.69	Ad
*S. dysenteria* CIP 5451	2.32	I	0.17	S	0.35	S

^A^ : *Lippia multiflora*; ^B^ : *Mentha piperita*; ^C^ : *Ocimum basilicum*; S. synergism; Ad. addition; I. indifference; ND. non determined

Only the combinations of *O. basilicum* with *L. multiflora* had synergetic effects against *E. aerogenes* and *S. enteric*, while *O. basilicum* in combination with *M. x piperita* had useful synergetic effects against *S. aureus*. When considering the number of synergetic effects by paired combination of EOs, the greatest number was obtained with combinations involving EO of *O. basilicum*.

The FIC indices of the associations of major components are reported in Tables 4. All the paired combinations involving eugenol showed synergistic effects on four bacteria, except its combinations with carvacrol and thymol against *P. aeruginosa* and *E. aerogenes*, respectively. The combinations containing thymol exhibited synergistic effects on the inhibition of *L. monocytogenes* and *P. aeruginosa* growth. The associations with carvacrol were synergistic against *E. aerogenes* and *E. coli*. Eugenol revealed stronger synergistic effect when combined with linalool and menthol. However, these three components alone have the weakest antibacterial activity when comparing to thymol and carvacrol (Table 2). Carvacrol and thymol showed a selective synergistic potential. No synergistic effects have been observed between menthol and linalool.

**Table 4:** Fractional inhibitory concentration and interaction between essential oil major components.

Components	*L. monocytogenes* CRBIP 13.134		*E. aerogenes* CIP 104725		*E. coli* CIP 105182		*P. aeruginosa* CRBIP19.249	
	FIC	Interaction	FIC	Interaction	FIC	Interaction	FIC	Interaction
Carvacrol/Eugenol	0.18	S	0.17	S	0.17	S	0.52	Ad
Carvacrol/Thymol	0.25	S	0.30	S	0.30	S	0.42	S
Carvacrol/linalool	3.05	I	0.17	S	0.16	S	2.02	I
Carvacrol/Menthol	3.05	I	0.17	S	0.18	S	2.07	I
Menthol/thymol	0.15	S	1.93	I	1.65	I	0.21	S
Menthol/Eugenol	0.06	S	0.04	S	0.09	S	0.31	S
Menthol/linalool	0.79	Ad	1.45	I	048	Ad	0.78	Ad
Eugenol/Thymol	0.17	S	1.52	I	0.17	S	0.09	S
Eugenol/linalool	0.05	S	0.04	S	0.06	S	0.03	S
Thymol/linalool	0.15	S	1.53	I	1.59	I	0.20	S

The efficacy of combinations appears to be related to chemical composition of combined EOs and to possible interactions between their major components. The increases of the inhibitory capacity of EOs and terpene mixtures caused by different combinations between them have been described by several groups [14,15,32]. Gallucci *et al.* [15] pointed out the synergistic effects on the growth inhibition of *S. aureus* and *B. cereus* of menthol/thymol and thymol/eugenol combinations. A synergistic effect of the combination of cinnamon and clove for the inhibition of *L. monocytogenes, B. cereus* and *Yersinia enterocolitica* has been reported by Goñi et *al*. [32]. This last author has reported synergetic effects against *E. coli* of thymol/eugenol, carvacrol/eugenol and thymol/carvacrol combinations. The best synergetic effects among EOs and majors components were obtained with combinations involving *O. basilicum* EO and eugenol, confirming the role of certain components in the interaction.

It is noteworthy that although the antibacterial ability of eugenol, menthol and linalool were the weakest compared with the other two components, they also produced the lowest FIC index, revealing that the antibacterial activities of a single compound does not determine antibacterial activities of the combination of compounds. Similar results have been reported for eugenol and menthol by Pei *et al.* [14].

The interaction (synergy, antagonism or addition) between two compounds depends on the concentrations of the single component [32] and the overall susceptibility of the target microorganism [4]. This may explain variation of interaction observed between combinations and strains. The difference in activity between combinations of carvacrol and thymol with others could be due to the relative position of the hydroxyl group [27]. Pei *et al.* [14] hypothesized that the synergistic effects of eugenol/carvacrol and eugenol/thymol might be engendered by that carvacrol and thymol disintegrated the outer membrane of *E. coli*, making it easier for eugenol to enter the cytoplasm and combine with proteins. Based on Pei *et al.*’s [14] hypothesis, we suggest that the synergism could be due to the increase of one of three factors which determine the antimicrobial property of monoterpenes: their lipophilic properties, the potency of their functional groups and their aqueous solubility by either compound of a paired combination [33,34]. The absence of synergistic effect observed between the two monoterpene alcohols linalool and menthol lacking an aromatic ring suggests that the aromatic ring may significantly contribute to synergism.

The present study has demonstrated the potential of the combination of *L. multiflora, M. x piperita* and *O. basilicum* EOs to increase antibacterial activity. The best synergistic effects among EOs and majors components were obtained with combinations involving *O. basilicum* and eugenol respectively. Results also showed that one or more synergistic components can produce the desired antibacterial effect. 

## Experimental

### Plant materials and chemicals

*L. multiflora* (leaves), *M. x piperita* (stems and leaves) and *O. basilicum* (leaves) were collected in June 2009 from the botanical garden at the Institut de Recherche en Sciences Appliquées et Technologies (12°25'470’’ N latitude and 1°29'251’’ W longitude), Ouagadougou, Burkina Faso. Plants were identified at the Laboratoire de Biologie et d’Ecologie Végétale (Université de Ouagadougou), where a voucher specimen is deposited. The plant material used for the isolation of the essential oil was air-dried at room temperature. Carvacrol, eugenol, linalool, menthol and thymol were purchased from Sigma–Aldrich Chemie (Steinheim, Germany). The level of purity was ≥ 98%.

### Extraction of essential oils

Fractions of each dried plant material (200 g) were submitted to hydrodistillation using a Clevenger-type apparatus for 3 h. Anhydrous sodium sulphate was used to remove water after extraction. EOs were stored in airtight containers in a refrigerator at 4 °C. The yields were calculated according to the weight of the plant material before distillation (expressed in percent, w/w of the dry vegetable material). Five microliters of EO were diluted with dichloromethane (1 mL) containing of biphenyl (0.1 mg/mL, Merck, Germany) as internal standard, prior to GC-FID and GC-MS analyses.

### GC and GC/MS analyses

Gas chromatographic analysis was performed on an Agilent 6890N instrument equipped with a flame ionization detector and a DB-5 narrow bore column (length 10 m × 0.1 mm ID, 0.17 µm film thickness; Agilent, Palo Alto, CA, USA). Helium (average velocity 42 cm/s) was used as carrier gas and the oven temperature programme was: 60–165 °C (8 °C/min) and 165–280 °C (20 °C/min) with 1min post run at 280 °C. Samples (1 µL) were injected at 260 °C front inlet temperature and the split ratio was 100:1. Calculation of peak area percentage was performed on the basis of the FID signal using the GC HP-Chemstation software (Agilent Technologies). The GC-MS (HP 6890 coupled to HP 5972 MSD; Hewlett Packard, Palo Alto, CA, USA) was equipped with a ZB-5MS Zebron capillary column (length 30 m × 0.25 mm ID, 0.25 µm film thickness; Agilent). Helium (average velocity 39 cm/s) was used as carrier gas and the oven temperature was hold 45 °C for 2 min and increased from 45–165 °C (4 °C/min), 165–280 °C (15 °C/min. Samples (1 µL) were injected at 250 °C and the split ratio was 50:1. 

### Identification of components

The constituents were identified by comparison of their retention indices with those of the literature. The retention indices were determined in relation to a homologous series of *n*-alkanes (C_8_–C_32_) under the same operating conditions. Further identification was made by comparison of their mass spectra with those stored in NIST library or with mass spectra from literature [35,36]. Component relative percentages were calculated based on GC peak areas without using correction factors.

### Antibacterial assays

#### Microbial strains

The microorganisms used were: *Escherichia coli* CIP 105182, *Enterobacter aerogenes* CIP 104725, *Enterococcus faecalis* CIP 103907, *Listeria monocytogenes* CRBIP 13.134, *Pseudomonas aeruginosa* CRBIP19.249, *Salmonella enterica* CIP 105150, *Salmonella typhimurium* ATCC13311, *Shigella dysenteriae* CIP 54.51 and *Staphylococcus aureus* ATCC 9144.

#### Disc diffusion tests

The agar disk diffusion method was employed for the screening of antimicrobial activities of the EOs and their major components. The test was performed in sterile Petri dish (90 mm diameter) containing solid and sterile Mueller-Hinton agar medium (Becton Dickinson, USA). The EOs or their components, absorbed on sterile paper discs (5 μL per Whatman disc of 6 mm diameter), were placed on the surface of the media previously inoculated with 100 μL of overnight microbial suspension (10^8^ CFU/mL).

One filter paper disc was placed per Petri dish in order to avoid a possible additive activity. Every dish was sealed with laboratory film to avoid evaporation, then incubated aerobically at either 30 °C or 37 °C according to bacteria for 18 to 24 h, followed by measurement of the zone diameter of the inhibition expressed in mm. Antibiotic discs of erythromycin (15 µg/disc) and tetracycline (30 UI) were used as positive controls.

#### Micro-well dilution assay

The minimal inhibition concentration (MIC) values were studied for the bacterial strains which were sensitive to the EO and/or their major components in disc diffusion assay. Minimal inhibition concentration (MIC) values were determined using micro-well dilution assay method. A serial doubling two fold dilution of either EO or major component was prepared in a microtiter tray over the range 10 mg/mL–0,075 mg/mL in 100 µL Muller-Hinton broth. The broth was supplemented with ethanol absolute at 0.5% in order to enhance EOs solubility.

Overnight broth cultures of each strain were prepared from 18 h broth cultures and suspensions were adjusted to 0.5 McFarland standard turbidity. An aliquot of 100 µL of the inoculum was added to diluted EO. The final volume in each well was 200 µL. The plate was covered with a sterile plate sealer. Positive and negative growth controls were included in every test. The tray was incubated aerobically at either 30 °C or 37 °C according to bacteria for 18 to 24 h. The MIC is defined as the lowest concentration of the EO at which the microorganism tested does not demonstrate visible growth in the broth. Bacterial growth was indicated by turbidity. 

### Interaction studies using checkerboard method

The checkerboard method was performed using 96-well microtitre plates as described previously [6], to obtain the FIC (Fractional inhibitory concentration) index. The microplate assay was arranged as follows: EOA was diluted two-fold along the x-axis, whilst EOB was diluted two-fold along the y-axis. The final volume in each well was 100 µL comprising 50 µL of each EO dilution. Subsequently, 100 µL of media containing 2 × 10^6 ^CFU/mL of the indicator strain were added to all wells. The plates were then incubated at 30 °C or 37 °C for 18 h. The FIC indices were calculated as FICA + FICB, where FICA and FICB 18 are the minimum concentrations that inhibited the bacterial growth for EOs A and B, respectively. Thus, FICs were calculated as follows: FICA = (MICA combination / MICA alone) and FICB = (MICB combination / MICB alone). The results were interpreted as synergy (FIC < 0.5), addititive (0.5 ≤ FIC ≤ 1), indifference (1 < FIC ≤ 4) or antagonism (FIC > 4). All experiments were done in triplicate.

## Statistical Analysis

For comparison of MIC and FIC values, tests were made in triplicate. Analysis of variance was performed. Significant differences between means were determined by Fisher’s test at the threshold of (p < 0.05).
